# 

**Published:** 1882-08

**Authors:** 


ESTABLISHED IN 1855.

OLD SERIES, Vol. XX—No. 5. AUGUST, 1882. NEW SERIES Vol. I—No. 11

### ATLANTA

### MEDICAL REGISTER.

(*ATLANTA MEDICAL AND SURGICAL JOURNAL.*)

EDITED BY

JAMES B. BAIRD, M.D.

H. H. DICKSON, PUBLISHER. TERMS, $2.50 A YEAR IN ADVANCE

ATLANTA, GEORGIA:

*H. H. DICKSON, BOOK AND JOB PRINTER AND BOOKBINDER.*

1882.

